# Highly Sensitive CH_4_/C_2_H_2_ Dual-Component TDLAS Sensor Based on a Dual-Channel Hexagram Multi-Pass Cell

**DOI:** 10.3390/s26041267

**Published:** 2026-02-15

**Authors:** Xinyu Liang, Xiaorong Sun, Haiyue Sun, Runqiu Wang, Shunda Qiao, Ying He, Yufei Ma

**Affiliations:** 1 National Key Laboratory of Laser Spatial Information, Harbin Institute of Technology, Harbin 150001, China; 1iangxy2026@163.com (X.L.); sunxiaorong020228@163.com (X.S.); sunhaiyue282@163.com (H.S.); 15253997971@163.com (R.W.); shundaqiao@126.com (S.Q.); hearkenyi@163.com (Y.H.); 2 Zhengzhou Research Institute, Harbin Institute of Technology, Zhengzhou 450008, China

**Keywords:** tunable diode laser absorption spectroscopy (TDLAS), multi-pass cell (MPC), dual-component sensing

## Abstract

A tunable diode laser absorption spectroscopy (TDLAS) sensor with a highly sensitive dual-component for methane (CH_4_) and acetylene (C_2_H_2_) detection is reported in this paper for the first time. A multi-pass cell (MPC) design model was established employing a vector-based ray-tracing method. A dual-channel MPC with an interlaced dual hexagonal star pattern was designed to improve gas absorption and realize real-time synchronous detection of CH_4_ and C_2_H_2_. During the simultaneous continuous monitoring of CH_4_ and C_2_H_2_, the sensor exhibited an excellent linear response to concentration variations. The minimum detection limit (MDL) for CH_4_ reached 132.08 ppb, improving to 77.32 ppb when the average time was increased to 300 s. In the case of C_2_H_2_, the MDL was measured at 20.19 ppb and further reduced to 3.50 ppb under the same extended average time.

## 1. Introduction

Trace gas detection serves as a critical technology in a wide range of applications, spanning environmental monitoring, industrial processes, and healthcare systems [1,2,3,4,5,6,7,8,9]. Methane (CH_4_), which accounts for more than 85% of natural gas composition, is a primary contributor to mining explosions and acts as a potent greenhouse gas. Its global warming potential over a 20-year horizon is approximately 84 times greater than that of carbon dioxide [10,11,12]. In recent years, accelerated global industrialization has driven a consistent rise in atmospheric CH_4_ levels, increasing at an average annual rate of 15–17 ppb and posing substantial risks to global climatic stability [13,14,15]. Concurrently, acetylene (C_2_H_2_) represents a key feedstock in chemical synthesis for numerous organic compounds and is extensively used in metalworking applications such as cutting and welding. Nevertheless, it exhibits high flammability and explosivity, with an explosive range in air of 2.5% to 81%, where even minimal ignition sources may induce severe detonation [16,17]. Given the considerable implications of both gases for industrial safety and environmental oversight, there is a pressing need for sensor systems that enable simultaneous, high-sensitivity detection of CH_4_ and C_2_H_2_.

Among existing techniques for trace gas analysis, laser absorption spectroscopy (LAS) is distinguished by its high selectivity, sensitivity, and rapid response [18,19,20,21,22,23,24,25,26,27,28,29,30,31,32]. Commonly employed LAS methods include photoacoustic spectroscopy (PAS), quartz-enhanced photoacoustic spectroscopy (QEPAS), tunable diode laser absorption spectroscopy (TDLAS), and light-induced thermoelastic spectroscopy (LITES) [33,34,35,36,37,38,39,40,41,42,43,44,45]. PAS configurations typically require relatively large photoacoustic cells, which complicate miniaturization and system integration. Both QEPAS and LITES rely on quartz tuning forks as detectors, which are limited by narrow bandwidths and present optical alignment difficulties. In contrast, TDLAS is based on direct absorption and does not depend on photoacoustic resonance or quartz transducers, thereby avoiding modulation frequency constraints. Its optical layout allows straightforward alignment, and the interface between photodetectors and supporting electronics is comparatively simple to implement.

Since TDLAS quantifies gas concentration by measuring laser intensity attenuation due to absorption, the technique currently confronts two main challenges [46]. The first pertains to achieving high detection sensitivity within a compact footprint. According to the Lambert–Beer’s law, the strength of the absorption signal is proportional to the optical path length (OPL). Extending OPL traditionally increases system volume (V), which conflicts with the pursuit of miniaturized sensor designs. Thus, realizing a long OPL in a small form factor remains a significant hurdle in TDLAS development. Multi-pass cells (MPCs) are frequently utilized in TDLAS systems to enhance effective OPL and improve detection sensitivity. A commonly adopted metric for evaluating MPC performance is the optical path length-to-volume ratio (OPL/V). Compared with conventional MPC designs such as White and Herriott cells, MPCs with dense spot patterns offer superior OPL/V and have recently gained considerable research interest. The second challenge in TDLAS involves simultaneous multi-gas detection. Many reported studies employ diode lasers to scan adjacent absorption lines of different gases sequentially. However, this strategy is only viable for specific gas combinations and lacks broad applicability.

In this work, we proposed a high-sensitivity dual-component TDLAS sensor based on a novel MPC with an interlaced dual hexagonal star pattern for the first time. An MPC design model was established employing a vector-based ray-tracing method. Two independent optical paths were configured to form a dual hexagonal star spot pattern. Through spatial division multiplexing within the MPC, a dual-component sensor capable of simultaneously monitoring CH_4_ and C_2_H_2_ concentrations was successfully implemented.

## 2. The Design of Dual Path MPC with Interlaced Dual Hexagonal Star Pattern

**Figure 1:**
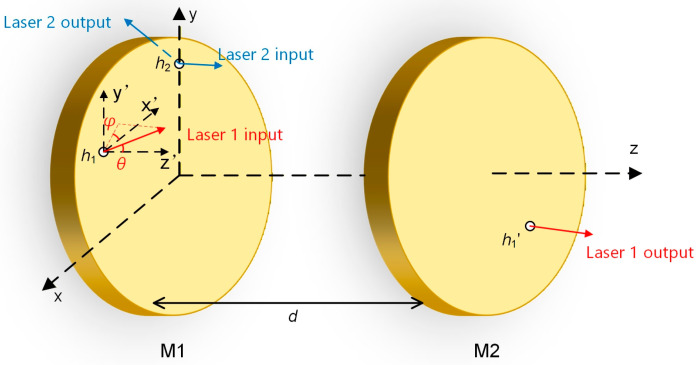
Structure diagram of the MPC. *d*: the distance between the mirrors; *θ*: the angle between the incident laser and the *Z* axis; *φ*: the angle between the projection of the incident ray in the *X*−*Y* plane and the *X* axis.

The Herriott cell is commonly employed owing to its straightforward construction and convenient optical alignment. Nevertheless, its typical circular or elliptical spot distributions yield a comparatively limited OPL/V [47,48,49]. To enhance the OPL/V, the laser incident angle can be increased, and spherical aberration induced by reflections from the concave spherical mirrors can be harnessed to generate a dense spot pattern using a dual-mirror configuration. Given that the laser enters the system at a non-paraxial angle, a vector-based ray-tracing methodology is appropriate for analyzing this MPC design. As illustrated in Figure 1, the self-designed MPC comprises two concave spherical mirrors, each with a radius of curvature of 100 mm and a diameter of 50.8 mm. Additionally, the surface quality of the mirrors is characterized by a peak-to-valley (PV) value of 3λ and an irregularity (IRR) of 0.5λ.

**Figure 2:**
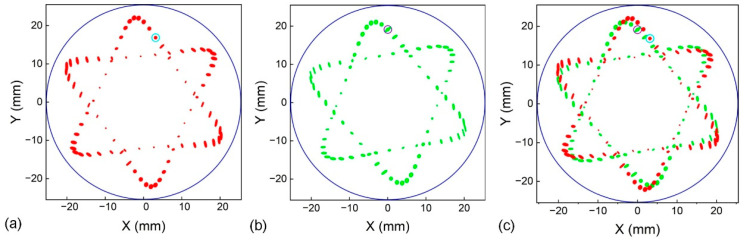
Simulated spot distribution of the MPC on the incident mirror. (**a**) Spot pattern of path 1. (**b**) Spot pattern of path 2. (**c**) Spot pattern of the double paths.

**Figure 3:**
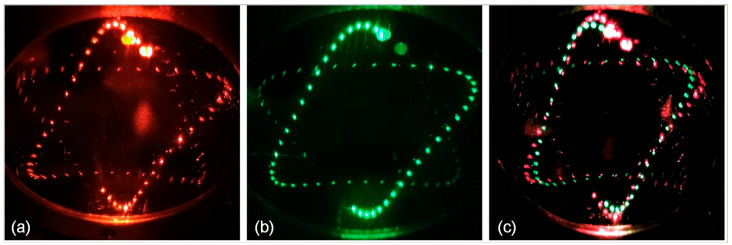
Measured spot distribution of the MPC on the incident mirror. (**a**) Spot pattern of path 1. (**b**) Spot pattern of path 2. (**c**) Spot pattern of the double paths.

The two mirrors are placed coaxially, with the optical axis defined as the *Z* axis. The distance between the two mirrors is denoted as *d*. Laser 1 and Laser 2 are simultaneously incident through different entrance holes *h* (*h*_1_ and *h*_2_) on Mirror 1 (M1). After hundreds of reflections, they exit the MPC through the exit hole *h*_1_′ on Mirror 2 (M2) and the entrance hole *h*_2_, respectively. The incident angles of Laser 1 and Laser 2 are defined by (*θ*_1_, *φ*_1_) and (*θ*_2_, *φ*_2_), respectively, where *θ* is the angle between the incident laser and the *Z* axis and *φ* is the angle between the projection of the incident ray on the *X*–*Y* plane and the *X* axis. In the vector-based ray-tracing method, the equations can be expressed as

(1) $$P_{i+1}=P_{i}+\rho_{i}\cdot {\vec{P}}_{i}$$

(2) $$\rho_{i}=\sqrt{(\vec{P_{i}}\cdot (P_{i}-r_{i}){)}^{2}-|P_{i}-r_{i}{|}^{2}+R^{2}}-{\vec{P}}_{i}\cdot (P_{i}-r_{i})$$

(3) $${\vec{P}}_{\left(i+1\right)}={\vec{P}}_{i}-2\;({\vec{P}}_{i}\cdot n_{i})\cdot n_{i}$$

where $P_{i}$ denotes the coordinate of the i-th spot, ${\vec{P}}_{i}$ represents the unit direction vector of the incident beam, and $n_{i}$ is the unit normal vector to the mirror surface at the i-th reflection. The $\rho_{i}$ corresponds to the geometrical path length of the i-th ray segment and the center of curvature for the mirror, denoted as $r_{i}$, which directs the i-th reflection. Using Equations (1)–(3) provided above, the coordinates of successive spots on the mirror can be computed iteratively. Figure 2a–c show the simulated spot patterns for the two individual optical paths and their combined dual-path configuration, respectively. Under a mirror separation of 104.94 mm, Laser 1 was directed into the cell at entrance hole *h*_1_ (3.13, 16.84) with incident angles *θ*_1_ at −9.25° and *φ*_1_ at −3.2°, generating the hexagonal star spot pattern shown in Figure 2a. The beam completed 167 reflections before emerging from exit hole *h*_1_′ (13.47, −12.14), producing an optical path length of 17.52 m. Given the MPC volume of 206 mL, the resulting OPL/V ratio reached 8.50 cm^−2^. Owing to the coaxial two-mirror configuration, rotating the spot pattern without altering its shape was achieved simply by adjusting the incident angles and entry location while retaining the original MPC geometry. As presented in Figure 2b, when the beam entered at entrance hole *h*_2_ (0, 19.12), it underwent 168 reflections before exiting through the same hole. This optical path was measured as 17.63 m in length and delivered an OPL/V ratio of 8.56 cm^−2^, with corresponding incident angles *θ*_2_ of −7.55° and *φ*_2_ of −7.45°. When both laser beams were simultaneously coupled into the MPC, an intricate interlaced dual-hexagonal-star spot pattern was obtained within the same compact volume of 206 mL, as shown in Figure 2c. Figure 3a–c present the corresponding experimentally recorded spot images, which are in good agreement with the simulation results. Both hexagonal-star spot patterns were formed by the aggregation of over 160 small reflection spots, which effectively prevents overlap interference and maximizes mirror utilization, thereby increasing the combined OPL/V of the dual optical paths to 17.06 cm^−2^.

In order to validate the discrepancy between the experimentally achieved OPL of the MPC and its theoretical design, as well as to examine potential optical crosstalk between the two independent beam paths, direct absorption experiments targeting CH_4_ and C_2_H_2_ were performed using the fabricated MPC. The measured spot distribution of the MPC on the incident mirror, obtained using fiber-coupled probe lasers, is illustrated in Figure 3. The detailed parameters of the MPC are listed in Table 1. The hole diameters on the mirrors were all set to 2 mm. The thickness of each mirror was 4 mm. The mirrors used were silver-coated, offering not only a reflectivity of 98% but also low cost and a broad operational wavelength range.

Compared to the MPCs reported in the existing literature, the present MPC achieves improvements in two critical parameters: OPL and OPL/V, demonstrating its superior performance. Table 2 provides a comprehensive comparison of MPCs from multiple current studies.

## 3. Experimental Setup

**Figure 4:**
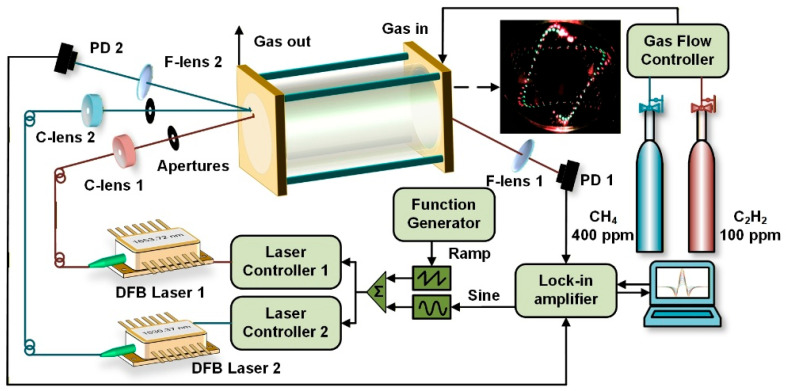
Experimental setup of the dual-component TDLAS sensor based on MPC with an interlaced dual hexagonal star pattern.

The experimental configuration is depicted in Figure 4, with insets displaying the actual spot patterns captured via red and green alignment lasers. After initial alignment, the fiber-coupled alignment lasers were substituted with fiber-coupled probe lasers without altering the position or orientation of the collimating lens (C-lens), thereby ensuring precise overlap between the probe beams and the intended optical paths. Two distributed feedback (DFB) diode lasers served as the optical sources for CH_4_ and C_2_H_2_ detection, respectively. The maximum output power of both lasers was approximately 15 mW. To suppress background noise, wavelength modulation spectroscopy (WMS) was implemented in this dual-gas sensor system. That is, the laser was modulated by superimposing a low-frequency sawtooth wave on a high-frequency sine wave. The modulated laser then sequentially passed through a collimating lens and pinhole apertures before entering the MPC. Following hundreds of reflections, an intricate interlaced hexagonal-star spot pattern emerged on the mirror surfaces. The two beams subsequently exited through separate output apertures and were converged onto the active areas of their corresponding photodetectors via focusing lenses (F-lenses) of 75 mm focal length. A 50 s ramp waveform was used to sweep the laser wavelength gradually across the target absorption line, while a 1 kHz sinusoidal modulation from a lock-in amplifier was simultaneously applied to each laser source. The detected signals were processed by the lock-in amplifier using second-harmonic (2*f*) demodulation with an integration time of 200 ms. Concentrations of CH_4_ and C_2_H_2_ inside the MPC were precisely regulated by adjusting the individual gas flow rates, while the total flow was held constant at 300 sccm.

**Figure 5:**
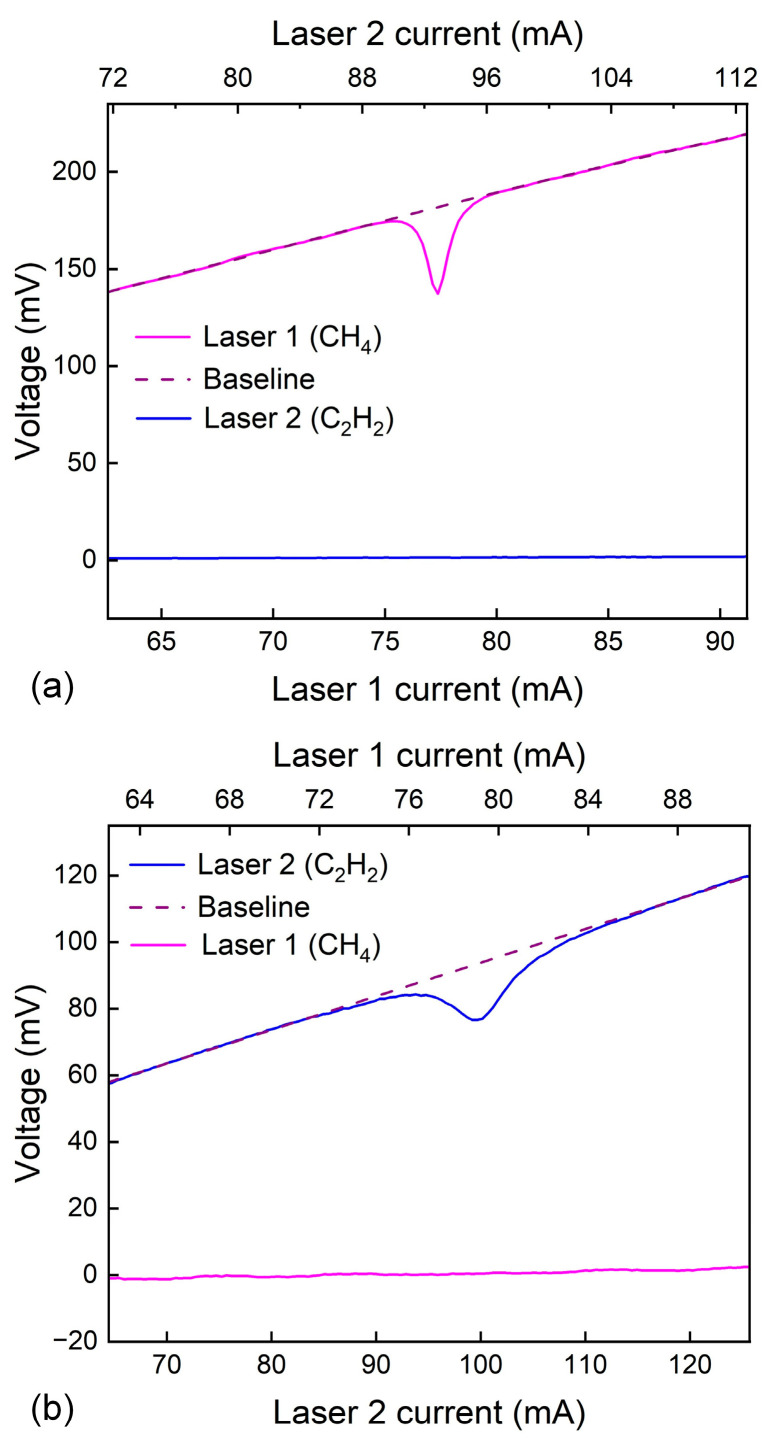
(**a**) Responses of PD1 to path 1 and path 2. (**b**) Responses of PD2 to path 1 and path 2.

**Figure 6:**
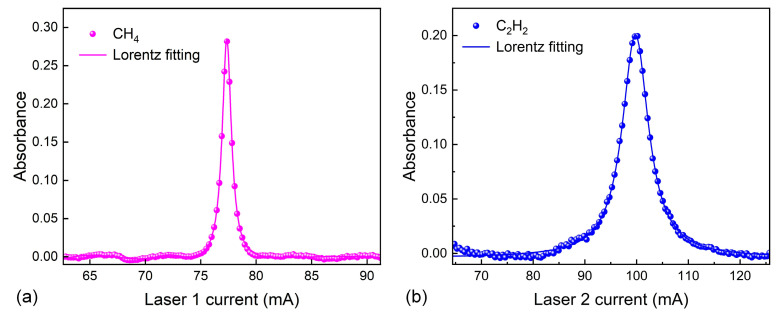
(**a**) The absorbance of 400 ppm CH_4_ at 6046.97 cm^−1.^ (**b**) The absorbance of 100 ppm C_2_H_2_ at 6534.37 cm^−1^.

Two absorption features were employed in this sensing system: one centered at 1653.72 nm (6046.97 cm^−1^) of CH_4_ and the other at 1530.37 nm (6534.37 cm^−1^) of C_2_H_2_. Measurements were carried out under near-standard atmospheric pressure and room temperature conditions. Within the MPC, the concentrations of CH_4_ and C_2_H_2_ were maintained at 400 ppm and 100 ppm. The corresponding results are presented in Figure 5. Scanning the injection current of Laser 1 across the CH_4_ absorption line yielded a detectable electrical signal exclusively on Photodetector 1 (PD1). Conversely, when the scan was applied to Laser 2, a pronounced signal appeared only on Photodetector 2 (PD2). A polynomial fit to the signal regions outside the absorption peaks provided the baseline reference. From the recorded absorption profiles and fitting baselines, absorbance values of 0.285 for CH_4_ and 0.201 for C_2_H_2_ were derived, with no appreciable crosstalk evident between the two channels. As illustrated in Figure 6, the measured OPLs for optical path 1 and optical path 2, calculated from the absorption data, were 18.91 m and 17.42 m, respectively. Observed deviations primarily stemmed from laser intensity drift and uncertainties in baseline fitting during signal processing.

## 4. Results and Discussion

**Figure 7:**
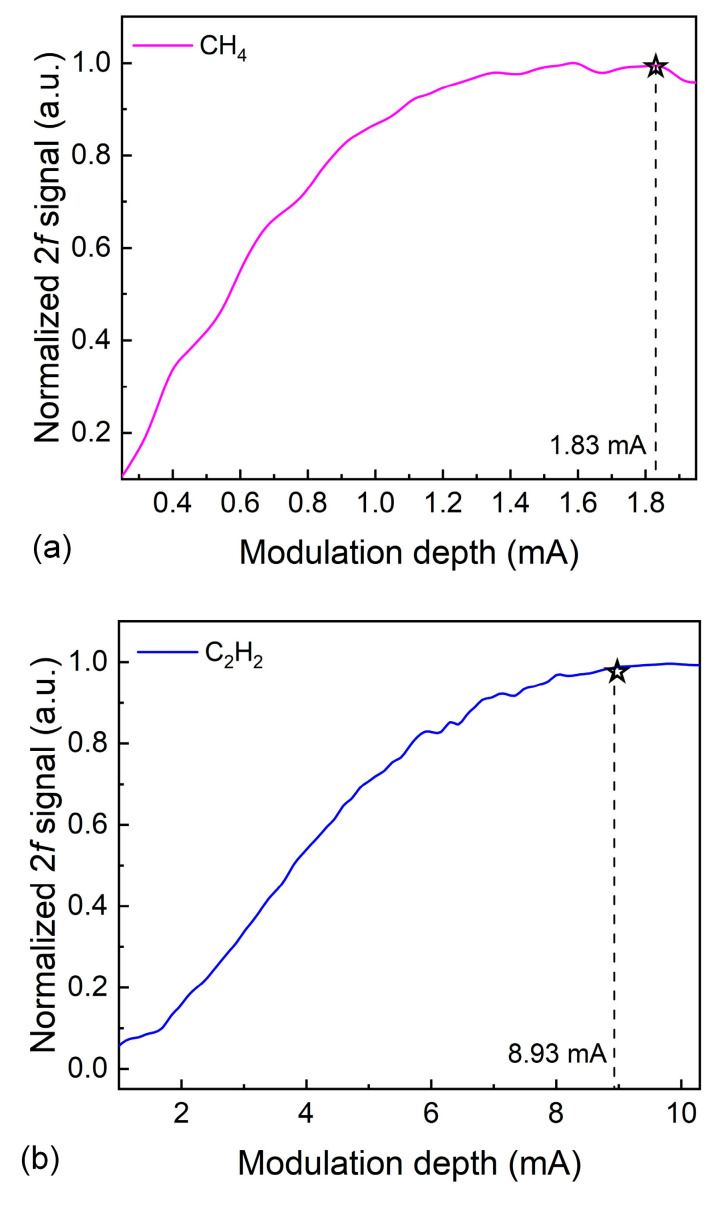
(**a**) Normalized 2*f* signal amplitude of 400 ppm of CH_4_ with different modulation depths. (**b**) Normalized 2*f* signal amplitude of 100 ppm of C_2_H_2_ with different modulation depths.

In this dual-gas sensor system, the concentrations of CH_4_ and C_2_H_2_ standard gases were set to 400 ppm and 100 ppm, respectively. The modulation depths for CH_4_/C_2_H_2_ dual-gas sensing were optimized, as shown in Figure 7. Optimal 2*f* signals for CH_4_ and C_2_H_2_ dual-gas sensing were obtained at modulation depths of 1.83 mA and 8.93 mA, respectively.

**Figure 8:**
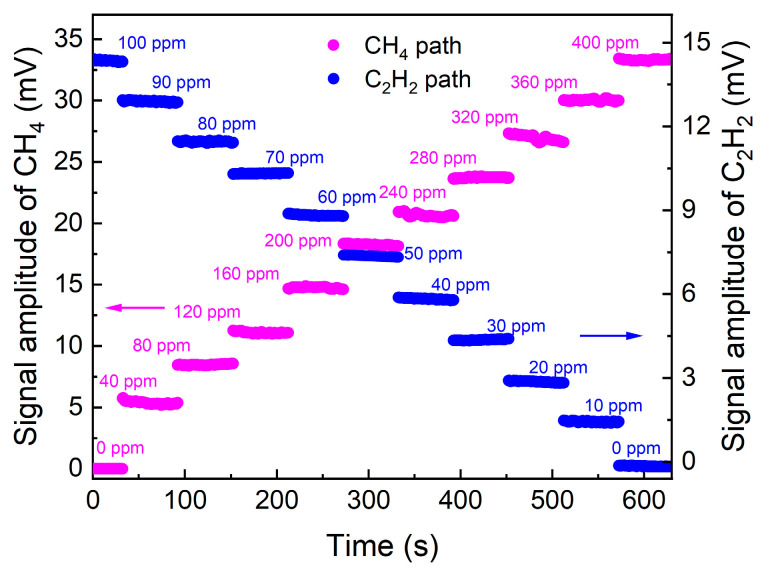
Concentration responses of the dual-gas TDLAS sensor based on MPC.

**Figure 9:**
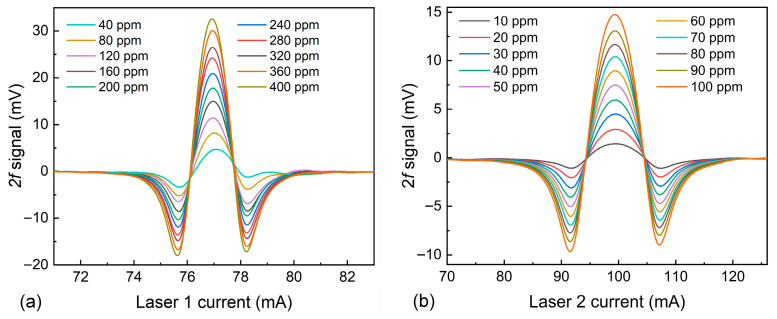
(**a**) 2*f* waveforms for CH_4_-TDLAS sensing. (**b**) 2*f* waveforms for C_2_H_2_-TDLAS sensing.

**Figure 10:**
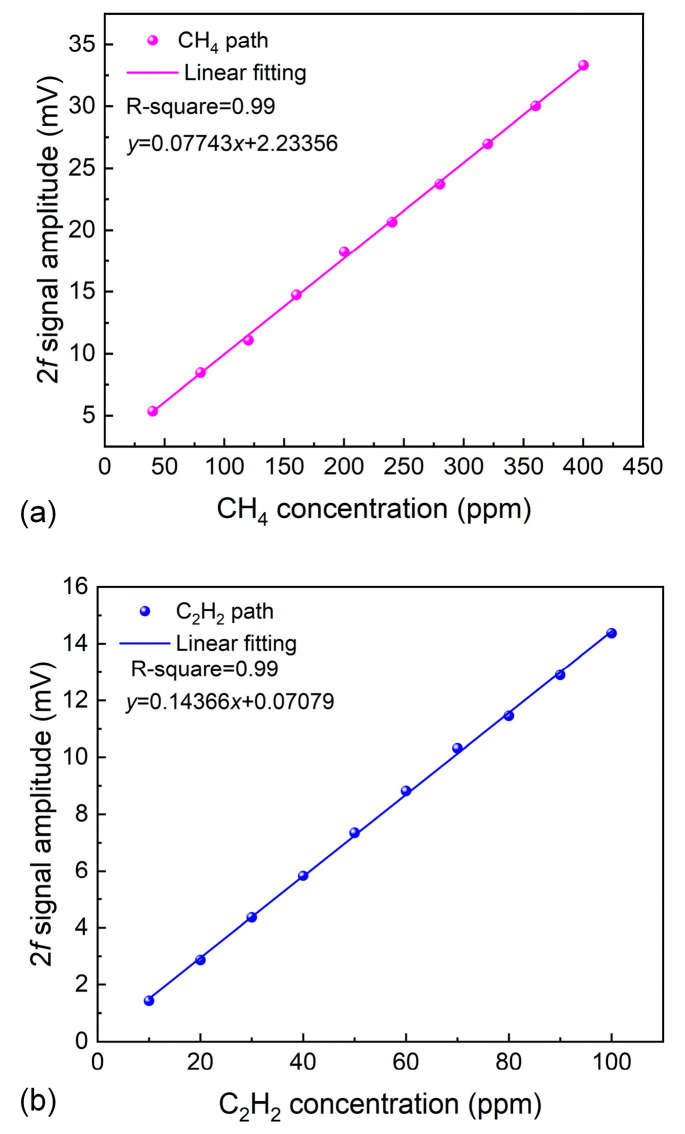
(**a**) The linear relationship between the 2*f* signal amplitude and concentration of CH_4_. (**b**) The linear relationship between 2*f* signal amplitude and concentration of C_2_H_2_.

The relationship between the 2*f* signal values and the concentrations of CH_4_ and C_2_H_2_ was investigated, with the results presented in Figure 8. Two mass flow controllers (MFCs) were used to adjust the flow rates of the CH_4_ and C_2_H_2_ standard gases, thereby regulating the concentrations of the dual-component gases in the MPC. The experimental results demonstrate that the 2*f* signals of CH_4_ and C_2_H_2_ TDLAS sensing were proportional to their respective concentrations, with no interference observed between the two gases. The 2*f* signal waveforms at different concentrations are displayed in Figure 9. The linear fitting of the concentration responses for CH_4_ and C_2_H_2_ was performed, as shown in Figure 10. After linear fitting, the R-squared values for both gas sensors were 0.99, indicating excellent linear responses of the sensor to concentration.

**Figure 11:**
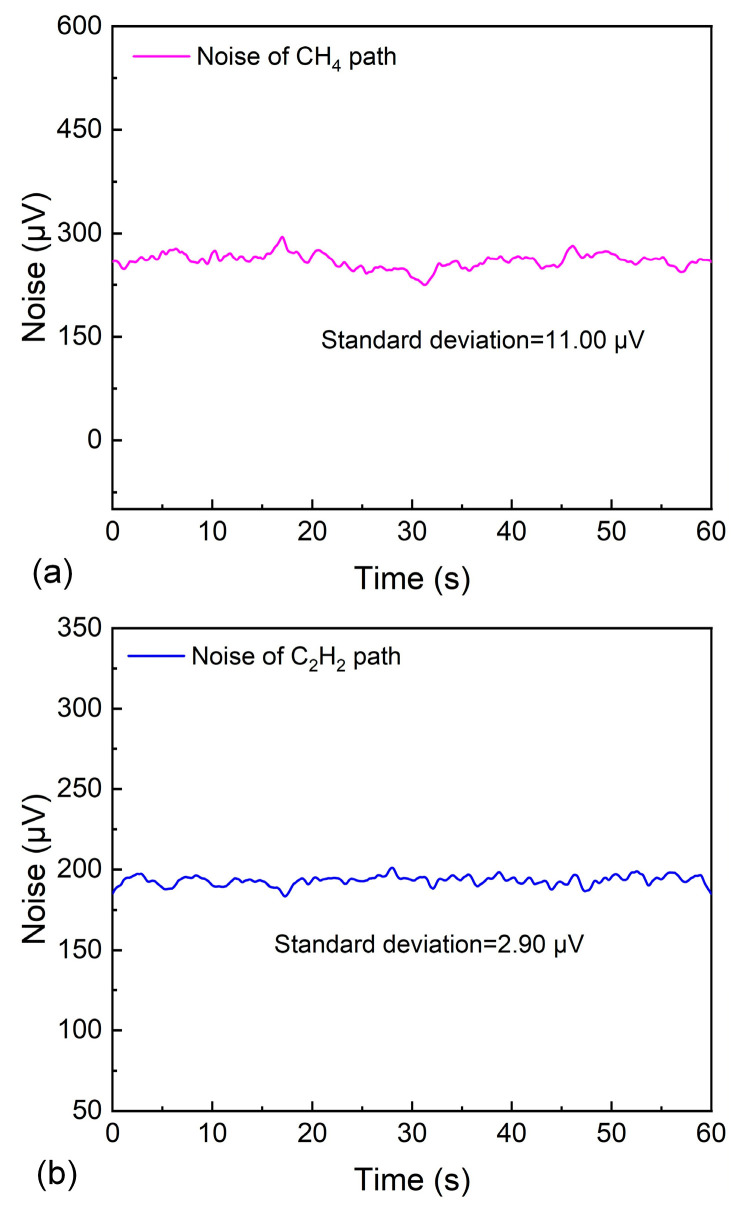
(**a**) Noise determination of CH_4_-TDLAS channel. (**b**) Noise determination of C_2_H_2_-TDLAS channel.

To determine the minimum detection limit (MDL), the background noise was measured by flushing the MPC with pure nitrogen (N2). As shown in Figure 11, the corresponding noise values for the two paths were 11.0 μV and 2.90 μV, respectively. Based on calculations, the MDLs for CH_4_ and C_2_H_2_ were determined to be 132.08 ppb and 20.19 ppb, respectively. The selected absorption line intensity of C_2_H_2_ is stronger than that of CH_4_, which enables significantly better detection performance for C_2_H_2_ compared to CH_4_ in the dual-gas TDLAS sensor.

**Figure 12:**
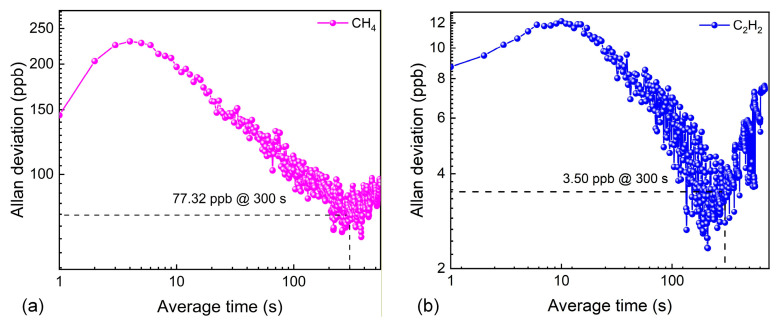
(**a**) Allan variance analysis of the CH_4_ channel. (**b**) Allan variance analysis of the C_2_H_2_ channel.

The long-term stability of the sensor system was evaluated using Allan deviation analysis. Pure N_2_ was flushed into the MPC. As shown in Figure 12, when the average time was extended to 300 s, the MDLs for CH_4_ and C_2_H_2_ were improved to 77.32 ppb and 3.50 ppb, respectively. Table 3 provides a comprehensive comparison of current spectroscopic techniques for detecting multiple gases. Clearly, the TDLAS system proposed in this work, utilizing an MPC with a dense interlaced dual hexagonal star pattern, offers the advantage of high-sensitivity simultaneous detection of CH_4_ and C_2_H_2_.

## 5. Conclusions

This paper presents a highly sensitive dual-gas TDLAS sensor for the simultaneous detection of CH_4_ and C_2_H_2_ based on a novel MPC with an interlaced dual hexagonal star pattern. A theoretical model for MPC design based on a pair of concave spherical mirrors has been developed. Within a compact volume of 206 mL, the MPC provides an OPL exceeding ten meters per channel, achieving an overall OPL/V of 17.06 cm^−2^ through its dense spot distribution. To further improve the sensor’s detection performance, WMS was implemented. The system exhibited excellent linear response for continuous, simultaneous monitoring of both target gases. The measured MDLs for CH_4_ and C_2_H_2_ were 132.08 ppb and 20.19 ppb, respectively. By extending the average time to 300 s, the MDLs could be further improved to 77.32 ppb for CH_4_ and 3.50 ppb for C_2_H_2_. Compared with conventional detection techniques, the proposed dual-component gas sensor offers notable advantages in sensitivity, compactness, and simultaneous multi-gas measurement capability, positioning it as a promising tool for applications in industrial safety and environmental monitoring.

## Figures and Tables

**Table 1:** Parameters of the MPC.

Variables	*d* (mm)	*h* (mm)	*θ*, *φ* (°)	N	OPL (m)	V (mL)	OPL/V (cm^−2^)
path 1	104.94	(3.13, 16.84)	(−9.25, −3.2)	167	17.52	206	8.50
path 2	104.94	(0, 19.12)	(−7.55, −7.45)	168	17.63	206	8.56

**Table 2:** Parameters of MPCs in different studies.

MPC	OPL (m)	OPL/V (cm^−2^)
Herriott cell [50]	6	0.17
TMPC [51]	1.9	3.74
CF-MPC [52]	4.85	3.57
This paper	35.15	17.06

**Table 3:** Performance comparison between different spectroscopy techniques.

Technology	Muti-Gas Type	MDL
PAS [53]	CH_4_	250 ppb @ 100 s
	C_2_H_2_	77 ppb @ 100 s
QEPAS [54]	CH_4_	730 ppb @ 100 s
	C_2_H_2_	1600 ppb @ 100 s
TDLAS (this paper)	CH_4_	77.32 ppb @ 300 s
	C_2_H_2_	3.50 ppb @ 300 s

## Data Availability

The data presented in this study are available upon request from the corresponding author.
